# Supplementary material for: Stability analysis and novel solutions to the generalized Degasperis Procesi equation: An application to plasma physics
Source: PLoS One. 2021 Sep 28;16(9):e0254816. doi: 10.1371/journal.pone.0254816 (PMC8478183; doi:10.1371/journal.pone.0254816)
Supplement: Attachment — Submitted filename: Response to Reviewer 2.pdf [file pone.0254816.s001.pdf]

Response to Reviewer2: (PONE-D-21-15906)

We sincerely and genuinely thank a referee for his/her valuable comments, which improved the manuscript in the present form. The concerns in the review report have been taken seriously and the manuscript has been revised accordingly.

- *We would like to thank the reviewer sincerely for this/her guidance, which will improve our manuscript. All the references that were suggested by the reviewer were discussed in the introduction and in other places in the text due to their strong connection to the topic under study.*
- *The whole manuscript has been revised and English-speaking professor corrects the language errors. We have done our best to improve our manuscript based on the referee's suggestions.*
- *In addition, the reference list has been updated.*

Again, we are grateful to a referee for his useful comments to improve our manuscript and we hope that the manuscript becomes suitable for publication in the present form.
